# Evaluating the Influence of Weight Status and Gender on Moderate to Vigorous Physical Activity and Sedentary Time in Physical Education Lessons: A Cross-Sectional Study

**DOI:** 10.3390/healthcare13050562

**Published:** 2025-03-05

**Authors:** Badriya Al-Hadabi, Meral Kucuk Yetgin, Fatih Dervent, Osama Aljuhani

**Affiliations:** 1 Physical Education and Sport Sciences Department, College of Education, Sultan Qaboos University, Muscat 123, Oman; bhaddabi@squ.edu.om; 2 Department of Coaching Education Sport Health Sciences, Faculty of Sport Sciences, Marmara University Sports Health Sciences, Istanbul 34815, Türkiye; meral.kucukyetgin@marmara.edu.tr; 3 Department of Applied Health Science, School of Public Health, Indiana University, Bloomington, IN 47405, USA; 4 Department of Physical Education Teacher Education, Faculty of Sport Science, Marmara University, Istanbul 34815, Türkiye; 5 Department of Physical Education, College of Sport Sciences and Physical Activity, King Saud University, Riyadh 11451, Saudi Arabia

**Keywords:** accelerometer, childhood obesity, MVPA, physical activity, physical education, sedentary

## Abstract

Background/Objectives: Low physical activity (PA) and excessive sedentary time negatively impact health, contributing to obesity. Physical education (PE) can help reduce the risk of obesity in schoolchildren. This cross-sectional study objectively measured moderate-to-vigorous physical activity (MVPA) and sedentary time during PE in Türkiye, examining the influence of weight status and gender. Method: Accelerometers measured MVPA in 274 children and adolescents (ages 10–14, 49.6% girls). Sedentary time and MVPA were classified based on accelerometer count per 15 s (sedentary: <25; MVPA: ≥574). A two-way Analysis of Covariance (ANCOVA) assessed body mass index (BMI) and gender effects on MVPA and sedentary time. Results: Students spent 44.5% of PE time sedentary and 43.2% in MVPA, below the recommended 50% of PE in MVPA. Only 45.6% met this target (44.2% males and 47.1% females). Overweight/obese students had higher sedentary time, while normal-weight students engaged in more MVPA (*p* < 0.05). Conclusions: Students did not meet the recommended 50% of PE in MVPA. Increasing MVPA and reducing sedentary time during PE is particularly important for overweight/obese students. Physical education intervention should target students in co-educational classes.

## 1. Introduction

Obesity is a major health concern affecting children and adolescents in many low- and middle-income countries [1,2]. Childhood obesity is a significant risk factor for developing cardiovascular complications in adulthood, including hypertension, diabetes, metabolic syndrome, and dyslipidemia [3,4]. Similarly, insufficient physical activity (PA) and sedentary behavior in children and adolescents are linked to various health issues [5,6,7,8]. For instance, prolonged screen time has been associated with increased adiposity [9], cardiometabolic risk factors, lower fitness, and reduced self-esteem [5]. Consequently, obesity treatment strategies often focus on promoting PA and reducing sedentary time [1]. Research indicates that, in Türkiye, the prevalence ratio of overweight and obesity increased between the 2000–2004 and 2010–2019 periods [2]. The World Health Organization (WHO) 2020 guidelines recommend that children and adolescents (ages 5–17) engage in at least 60 min of moderate-to-vigorous physical activity (MVPA) daily to achieve health benefits [10]. However, recent studies show that 50% of boys and 65% of girls in Türkiye are physically inactive [11]. Additionally, international research highlights that MVPA levels in children often fall below the recommended guidelines [12,13].

Children and adolescents spend approximately 5 to 6 h daily in school, making it a key environment for promoting health-related behaviors, such as increasing PA and reducing sedentary time [14]. Among school-based interventions, physical education (PE) lessons play a crucial role in enhancing PA levels [15]. Previous reviews found that targeted efforts to increase MVPA during PE led to higher PA levels [16] and reduced sedentary time outside school hours [17,18,19,20,21]. Moreover, evidence suggests that daily PE lessons in childhood have a long-term positive impact on health-related behaviors in both men and women [22].

The Center for Disease Control and Prevention (CDC) in the United States recommends that 50% of PE time be spent in MVPA [23]. Similarly, the British Association for Physical Education (AfPE) suggests that 50–80% of PE time should involve PA [24]. Extensive research has examined whether these recommendations are met. For instance, a systematic review found that when MVPA was measured using accelerometers, students engaged in MVPA for an average of 32.6% (ranging from 5.9% to 59.3%) of PE time [25]. Another accelerometer-based study reported that Saudi boys spent only 22% of PE time in MVPA [17]. In Türkiye, an observational study indicated that children engaged in 39.8% of PE time in MVPA [26]. Furthermore, a meta-analysis found that elementary school children spent 35.9% of PE time being sedentary [27].

Evidence suggests that overweight and obesity in childhood often persist into young adulthood. For instance, a study found that 62% of 841 young adults who were in the highest body mass index (BMI) quartile during childhood remained in that quartile as young adults [28]. Similarly, a review indicated that 42–63% of obese children continue to be obese in adulthood [29]. Research on the relationship between weight status and PA levels during PE has produced mixed findings. Studies suggest that overweight and obese children tend to spend less time in MVPA and more time being sedentary during PE [30]. Additionally, research indicates that overweight and obese students engage in lower levels of PA, which may increase health risks and negatively impact overall fitness [31,32,33]. However, some studies report no significant differences in MVPA and sedentary time between normal-weight and overweight/obese students [34,35]. Children with higher BMI may struggle to meet recommended PA levels, emphasizing the need for adaptive strategies within PE programs. Notably, research suggests that an additional 60 min of PE per week can reduce the likelihood of obesity among schoolchildren [36].

The attitude of Turkish students toward PE lessons has been reported as positive across genders [37]. However, Turkish boys tend to show a stronger preference for sports such as soccer, basketball, and weightlifting compared to girls [37,38]. Despite this, previous research using observational measurement methods found that boys and girls exhibited similar levels of MVPA and sedentary time during PE lessons [26]. Conversely, other studies have reported that boys spend more time in MVPA and less time in sedentary than girls during PE [39,40,41].

In Türkiye, most research has focused on students’ attitudes toward PE participation, but few studies have examined MVPA and sedentary time. Additionally, most studies that have used objective measurement tools (e.g., accelerometers) to quantify MVPA and sedentary time have been conducted outside Türkiye. For educators and policymakers, accurately measuring MVPA and sedentary time in PE classes is essential to understanding PE’s contribution to children’s daily PA and overall health. Furthermore, there is limited research in Türkiye assessing MVPA and sedentary time during PE among children with different BMI levels and across genders. Understanding the effects of BMI and gender on children’s MVPA levels and sedentary time is crucial for optimizing PE class design. Therefore, this study introduces a novel approach by utilizing accelerometers to compare MVPA and sedentary time within a new cultural context, offering more accurate data on gender and weight-related disparities. The study aimed to objectively measure PA intensity during PE classes in Turkish schools using accelerometers and determine whether students achieve the recommended MVPA levels during PE. Additionally, it aimed to examine the influence of BMI and gender on MVPA and sedentary time during PE classes. We hypothesized that overweight/obese students would engage in less MVPA and more sedentary time than normal-weight students and that gender differences in PA levels would be observed.

## 2. Materials and Methods

### 2.1. Participants

The study sample included 274 students (138 boys, 136 girls) aged 10–14 years, recruited from three public primary schools in Istanbul, Türkiye. This age range was selected due to the documented decline in PA levels [42]. All students from these schools were invited to participate, and the study was conducted on a voluntary basis. Statistical power analysis was conducted using G*Power software (version 3.1.9.4) to determine the required sample size for a statistical power of 0.80, assuming a medium effect size (0.25) and an alpha error probability of 0.05. The analysis estimated a required sample size of 122 participants. However, the final sample size exceeded this threshold, enhancing the statistical power, reducing potential biases, and providing more robust analyses. Children without major health conditions affecting mobility were eligible to participate. Parental consent was obtained, along with research approval from the Istanbul Provincial Directorate of National Education and an ethics committee. The study was conducted in Bahçelievler Kumport Secondary School, Başakşehir Borsa Istanbul Alparslan Secondary School, and Küçükçekmece Hayriye Gök Secondary School. Data collection took place in April 2023.

### 2.2. Anthropometry

Height was measured using a portable, calibrated Seca 220 stadiometer (SECA, Hamburg, Germany) with a precision of 0.1 cm, while body weight was recorded using a digital scale with a precision of 0.1 kg. Students were instructed to remove their shoes and wear light clothing to ensure measurement accuracy. BMI was calculated using the standard formula BMI (kg·m^−2^), and age- and sex-specific BMI cut-off points [43] were used to classify the participants as normal-weight or overweight/obese. This classification method has been widely used in previous studies [38,40]. For this study, lean participants were grouped with normal-weight students.

### 2.3. MVPA and Sedentary Measures

Accelerometers are recognized as an objective tool for assessing MVPA levels and sedentary time in both school and non-school settings, including PE classes, break times, classroom sessions, and after-school periods [44]. During PE classes, accelerometers track movement patterns, enabling researchers to quantify time spent in MVPA and sedentary. This approach has been widely used in structured settings such as PE classes [17,30,38,45]. In this study, ActiGraph wGT3X-BT triaxial accelerometers (ActiGraph LLC, Pensacola, FL, USA) were used to assess MVPA and sedentary time during PE lessons. At the beginning of each class, students wore the accelerometers on their right hip using an elastic belt. After the lesson, they removed the devices and returned them to the researchers. PE teachers adhered to a pre-approved study plan to ensure consistency in PE instruction. Accelerometer devices were initialized, downloaded, and analyzed using ActiLife v6.13.6 software (ActiGraph LLC, Pensacola, FL, USA). The data were stored in AGD file format with a 15-s epoch. Validated accelerometer cut-off points were applied to classify activity levels: sedentary time (<25 counts) and MVPA (≥574 counts) [46]. Analyzed data were exported to Microsoft Excel 2019, where time in minutes was converted into decimal values for ease of statistical processing.

### 2.4. Data Analysis

Descriptive statistics were calculated to summarize the data, including mean (± SD), frequencies, and percentages. Since lesson durations varied, MVPA and sedentary time were expressed as percentages to ensure comparability. To examine the influence of BMI and gender on MVPA and sedentary time, two separate two-way Analyses of Covariance (ANCOVA) were performed. These analyses assessed the main effects of BMI (overweight/obese vs. normal-weight), gender (male vs. female), and the interaction effect (BMI × gender) on MVPA and sedentary time, with age included as a covariate. The normality of residuals was visually inspected using histograms, followed by the Shapiro–Wilk test. Levene’s test confirmed that the assumption of homogeneity of variances was met (all *p* > 0.5). All statistical analyses were conducted using the Statistical Package for the Social Sciences (SPSS) v28.0, with statistical significance set at α < 0.05.

## 3. Results

Table 1 presents the descriptive statistics for the 274 participants. The mean age of the participants was 12 ± 1.0 years, with 66 students (24%) classified as overweight or obese and 136 students (49.6%) identified as female. The percentage of time spent in sedentary and MVPA during PE lessons was 44.5% and 43.2%, respectively. On average, normal-weight male and female students engaged in a similar proportion of MVPA. However, overweight/obese female students spent the highest proportion of time in sedentary (52.3%).

Table 2 presents the number and percentage of students who met the recommended 50% PE time in MVPA. The data indicate that 47.8% of normal-weight males and 50.8% of normal-weight females met the target. Among overweight/obese students, 37.8% of males and 22.2% of females achieved the recommendation.

The results of the two-way ANCOVA are presented in Table 3. A significant main effect of the BMI group was observed for sedentary time (F (1, 269) = 7.092, *p* = 0.008, partial η^2^ = 0.026) and MVPA (F (1, 269) = 4.692, *p* = 0.031, partial η^2^ = 0.017. However, no significant main effect of gender was found for time spent in MVPA or sedentary. Additionally, the results showed no significant interaction effect between the BMI group and gender for sedentary time or MVPA. Pairwise comparisons revealed that normal-weight participants spent a greater percentage of PE time in MVPA (mean difference: 5.7, 95% CI = 0.5, 10.7), whereas overweight/obese participants spent more time in sedentary (mean difference: 7.8, 95% CI = 2.0, 13.6).

## 4. Discussion

To the best of our knowledge, this is the first study to objectively measure sedentary time and MVPA levels using accelerometry during PE lessons in Turkish schools. This cross-sectional study aimed to assess MVPA and sedentary time during PE and examine gender- and weight-related differences. The results showed that students spent 44.5% and 43.2% of PE time in sedentary and MVPA, respectively. Additionally, sedentary time was higher among overweight/obese participants, whereas MVPA levels were greater among normal-weight participants. Both male and female students spent a similar proportion of PE time in MVPA and sedentary.

Regarding the first objective, the proportion of PE time spent in MVPA in this study was approximately 10% higher than in previous accelerometer-based studies that examined MVPA during PE [25,27]. This higher MVPA proportion may be attributable to PE content, as prior research suggests that intensive PE activities, such as team games and fitness training, can increase MVPA levels [40]. Furthermore, the mean lesson duration in this study was 60.3 min (range: 35–90 min). Longer PE lessons (e.g., 60–90 min) have been associated with a higher percentage of MVPA [47]. Future intervention studies should examine how lesson duration and content influence MVPA levels. Other teacher-related factors may also play a role. For instance, teachers with less than five years of experience tend to spend more time on class management [48]. Additionally, PE lessons led by female teachers have been associated with lower MVPA levels [48]. Meanwhile, the proportion of PE time spent in sedentary in this study was higher than that reported in Japan (27.3%) and Finland (37.3%) [45,47]. This relatively high sedentary time is concerning. One possible explanation is that class management practices influenced sedentary time. In Australia, a previous study found that approximately one-third (30.8%) of PE time was spent on organizational tasks and management [49]. Future interventions should focus on reducing sedentary time by incorporating more light-intensity activity while promoting MVPA during PE.

Unfortunately, the mean percentage of MVPA did not meet the recommended 50% of PE time spent in MVPA [23,24]. However, 45.6% of students achieved the recommendation, considerably higher than previous reports of 8%, 13.8%, and 26% [38,40,41]. Although the target was not fully met, these findings highlight the contribution of PE to daily MVPA. On average, students spent 25 min in MVPA during PE lessons, which equates to 42% of the daily recommended MVPA (60 min/day). Previous research found that each additional minute of MVPA during PE contributes to a 1.4-min increase in daily MVPA [38], reinforcing the potential role of PE in enhancing overall PA levels. However, caution should be exercised when comparing these results with previous accelerometer-based studies, as differences in accelerometer protocols (e.g., cut-points and wear location) may lead to variability in results. For example, research suggests that compliance with PE recommendations increases when accelerometer epoch lengths are extended [50].

Regarding the second objective, we found no differences in the mean percentage of MVPA and sedentary time between boys and girls. These findings align with previous studies that reported no gender differences when accelerometers were used to measure MVPA and sedentary time in PE lessons [45,47]. However, an Estonian study using accelerometers found significant gender differences in MVPA and sedentary time [38]. Additionally, another study reported that girls were more active in single-gender PE classes compared to co-educational classes [51]. Using a pedometer, Hannon et al. [52] found that boys were more active than girls in co-educational PE classes. The lack of significant gender differences in our study may be attributed to equitable opportunities for PA in co-educational PE lessons, where both genders participate under similar conditions [39]. Another possible explanation is that girls in this study may have had a positive attitude toward engaging in PE activities. A previous study reported that Turkish girls had a more positive attitude toward PE than boys [37]. However, caution is needed when interpreting this result, as attitudes toward PE may not fully explain gender-based differences in PA levels within structured settings like PE classes. Further research should examine students’ attitudes toward PE while simultaneously assessing their actual engagement in MVPA and sedentary during PE lessons.

In the current study, we also examined the influence of weight status on MVPA and sedentary time during PE. Previous studies have reported mixed and inconsistent findings. Our results showed that normal-weight students spent more time in MVPA and less time in sedentary than overweight/obese students. These findings are consistent with previous studies that found that normal-weight students engaged in significantly more MVPA and less sedentary time than their overweight/obese peers [30,53]. However, some studies reported no significant differences between normal-weight and overweight/obese students in MVPA and sedentary time during PE [34,35]. Notably, overweight/obese students in this study engaged in an average of 24 min of MVPA during PE lessons. This accounts for 40% of the daily recommended MVPA. This result suggests that PE has the potential to engage overweight/obese students in higher levels of MVPA. However, these findings should be interpreted with caution, as PE content and the type of PE class (e.g., single-gender vs. co-educational) were not examined in this study. These factors may influence PA and sedentary time differences between normal-weight and overweight students. Thus, further research is needed to explore the impact of PE structure, content, and instructional strategies on PA levels during PE lessons.

The strength of this study lies in its objective measurement of MVPA levels and sedentary time in a reasonably sized sample during PE lessons in Turkish schools. However, several limitations should be considered. First, the study did not observe the types of activities conducted during PE lessons. Previous research suggests that MVPA time in PE can vary depending on the activities being taught [15]. Although accelerometers are valid and reliable tools for measuring PA levels and sedentary time in children and adolescents, they may underestimate activities involving upper-body movement. Another limitation is that the study did not examine the relationship between the duration of sedentary bouts and children’s weight status. This aspect is particularly important given that long PE lessons (e.g., ≥60 min) may involve extended sedentary periods. Future longitudinal research should explore how prolonged sedentary bouts (e.g., ≥20 min) influence children’s weight status during PE. Additionally, MVPA and sedentary time were measured in co-educational PE lessons. A previous study found that boys and girls were more active in co-educational lessons than in boys-only PE classes [52]. However, the study did not investigate the influence of teacher behavior on students’ MVPA and sedentary time, despite prior research showing that teacher behavior significantly impacts students’ MVPA levels during PE [54]. Furthermore, while we found significant differences in MVPA and sedentary time between normal-weight and overweight/obese students, psychological factors may partially influence these findings. Previous studies report that overweight and obese children [55] tend to have lower physical self-esteem, which could affect their engagement in MVPA and sedentary time during PE lessons. Finally, this study was limited to schools in Istanbul, meaning the results may not be generalizable to other regions in Türkiye. Future research should objectively measure MVPA and sedentary time in PE lessons using a nationally representative sample to enhance generalizability.

## 5. Conclusions

The results of this study showed that Turkish students spent 44.5% and 43.2% of PE time in sedentary and MVPA, respectively. The proportion of MVPA time was below the recommended 50% of PE time. Overweight students spent more time in sedentary behavior, while non-overweight students engaged in higher levels of MVPA. No significant gender differences were found in MVPA and sedentary time percentages. Increasing MVPA and reducing sedentary time during PE is particularly important for overweight/obese students. Physical education intervention should target students in co-educational classes.

## Figures and Tables

**Table 1:** Descriptive statistics for study variables according to gender and BMI groups.

	Total		Male		Female	
	Overweight/Obese	Normal-Weight	Overweight/Obese	Normal-Weight	Overweight/Obese	Normal-Weight
	N = 66	N = 208	N = 48	N = 90	N = 18	N = 118
Age (years)	11.9 (1.1)	12.0 (1.0)	12.0 (1.1)	12.0 (1.0)	11.9 (1.1)	12.0 (1.1)
Height (cm)	153.9 (10.1)	151.9 (9.9)	155.6 (10.4)	151.4 (11.2)	149.4 (9.0)	152.1 (8.9)
weight (kg)	58.1 (11.6)	40.0 (7.9)	60.1 (11.4)	39.9 (8.5)	53.1 (10.9)	40.1 (7.5)
BMI (kg·m^−2^)	24.3 (2.6)	17.2 (2.1)	24.6 (2.6)	17.2 (2.1)	23.4 (2.6)	17.2 (2.2)
Proportion in Sedentary (%)	50.0 (19.8)	42.8 (18.5)	49.1 (19.8)	43.5 (19.6)	52.3 (20.1)	42.3 (17.7)
Proportion in MVPA (%)	39.9 (17.2)	44.3 (17.1)	41.5 (17.1)	44.2 (18.1)	35.7 (17.1)	44.4 (16.5)

Data are presented as mean (SD); BMI: body mass index; and MVPA: Moderate and Vigorous Physical Activity.

**Table 2:** Number (*n*) and percentage (%) of students achieved the recommendation of 50%PE time.

	Total		Male		Female	
	*n*	%	*n*	%	*n*	%
Normal-weight	103	49.5	43	47.8	60	50.8
Overweight/obese	22	33.3	18	37.8	4	22.2

**Table 3:** Two-way ANCOVA results for study variables.

	Main Effect						Interaction		
	BMI Group			Gender			BMI Group × Gender		
	F-Test	*p*	Partial η^2^	F-Test	*p*	Partial η^2^	F-Test	*p*	Partial η^2^
Sedentary	7.092	0.008	0.026	0.121	0.729	0.001	0.560	0.455	0.001
MVPA	4.692	0.031	0.017	1.146	0.285	0.004	1.351	0.246	0.005

All ANCOVA tests are adjusted for age. *p* < 0.05.

## Data Availability

All relevant data are available in the manuscript.
